# Current Advancements of Plant-Derived Agents for Triple-Negative Breast Cancer Therapy through Deregulating Cancer Cell Functions and Reprogramming Tumor Microenvironment

**DOI:** 10.3390/ijms222413571

**Published:** 2021-12-17

**Authors:** Tai-Na Wu, Hui-Ming Chen, Lie-Fen Shyur

**Affiliations:** 1 Agricultural Biotechnology Research Center, Academia Sinica, Taipei 115, Taiwan; taina@gate.sinica.edu.tw (T.-N.W.); hmchen@gate.sinica.edu.tw (H.-M.C.); 2 Ph.D. Program in Translational Medicine, College of Medicine, Kaohsiung Medical University, Kaohsiung 807, Taiwan; 3 Graduate Institute of Pharmacognosy, Taipei Medical University, Taipei 110, Taiwan

**Keywords:** triple-negative breast cancer, metabolism, tumor microenvironment, metastasis, drug resistance, phyto-adjuvants

## Abstract

Triple-negative breast cancer (TNBC) is defined based on the absence of estrogen, progesterone, and human epidermal growth factor receptor 2 receptors. Currently, chemotherapy is the major therapeutic approach for TNBC patients; however, poor prognosis after a standard chemotherapy regimen is still commonplace due to drug resistance. Abnormal tumor metabolism and infiltrated immune or stromal cells in the tumor microenvironment (TME) may orchestrate mammary tumor growth and metastasis or give rise to new subsets of cancer cells resistant to drug treatment. The immunosuppressive mechanisms established in the TME make cancer cell clones invulnerable to immune recognition and killing, and turn immune cells into tumor-supporting cells, hence allowing cancer growth and dissemination. Phytochemicals with the potential to change the tumor metabolism or reprogram the TME may provide opportunities to suppress cancer metastasis and/or overcome chemoresistance. Furthermore, phytochemical intervention that reprograms the TME away from favoring immunoevasion and instead towards immunosurveillance may prevent TNBC metastasis and help improve the efficacy of combination therapies as phyto-adjuvants to combat drug-resistant TNBC. In this review, we summarize current findings on selected bioactive plant-derived natural products in preclinical mouse models and/or clinical trials with focus on their immunomodulatory mechanisms in the TME and their roles in regulating tumor metabolism for TNBC prevention or therapy.

## 1. Introduction

Triple negative breast cancer (TNBC), defined by the absence of estrogen, progesterone, and human epidermal growth factor (HER) receptors, is a subtype of breast cancer which is remarkably aggressive, metastatic, and drug resistant. TNBC accounts for around 10.4% of total breast cancers and a disproportionate 83.3% of deaths in comparison with other hormone receptor-positive or HER2-positive subtypes of breast carcinomas [1]. About 25% of TNBC patients carry germline mutations of *BRCA1/2* [2]. *BRCA1* and *BRCA2* are involved in homologous recombination repair of double-stranded DNA (dsDNA) breaks, and mutational loss of *BRCA1/2* renders cells more susceptible to drugs that block other DNA repair mechanisms. The primary function of poly-ADP-ribosyl polymerase (PARP) is to repair single-stranded DNA breaks before they advance to double-stranded breaks [3]. PARP inhibitors have been developed to treat multiple cancer types with *BRCA1/2* mutations, thereby creating synthetic lethality in *BRCA1/2*-defective cells [4].

Approximately 20% of TNBC patients respond well to standard therapy (tumor resection, radiation, and cytotoxic chemotherapy), but the rest develop lethal metastatic disease. The current chemotherapeutics for TNBC including platinum, anthracycline, and paclitaxel tend to develop drug resistance due to their poor bioavailability and cellular uptake, off-target effects, and diverse/severe side-effects that have limited their usage in patients [5,6]. In addition, the occurrence of drug resistance of TNBC is associated with tumor heterogeneity with genomic alterations in the tumor suppressor genes or oncogenic drivers (e.g., *BRCA1/BRCA2*, *RB1, TP53, Phosphoinositide 3-kinases (PI3K)/Phosphatase and tensin homolog (PTEN), PIK3CA*) and the complexity of the tumor microenvironment (TME) [7,8]. Although druggable or direct targets for TNBC disease are still lacking, to overcome the treatment challenges, potential “targeted” therapies that deregulate immune checkpoints or specific molecular pathways activated in cancer cells are being explored [9].

TNBC has six major heterogeneous subtypes: basal like-1, basal like-2, immunomodulatory, mesenchymal, mesenchymal stem-like, and luminal androgen receptor [10]. The basal like-subtypes are characterized by defects in DNA repair pathways and cell-cycle checkpoints, which are sensitive to antimitotic and DNA-damaging agents (e.g., PARP inhibitors and platinum-based drugs) [11]. PARP inhibitors are synthetic, lethal with germline *BRCA* mutant breast cancer, and approved for HER2-negative and metastatic breast cancer therapy. Talazoparib and Olaparib were approved in 2018 by the U.S. Food and Drug Administration (FDA). Both mesenchymal and mesenchymal stem-like subtypes are enriched in genes involved in cell motility, extracellular matrix (ECM) receptor interaction and cell differentiation pathways; however, genes associated with stemness, mesenchymal stem cell-specific markers and angiogenesis, which are sensitive to inhibitors targeting the PI3K/AKT/ mTOR and Src pathways, are unique to the mesenchymal stem-like subtype [11,12]. The immunomodulatory subtype is fortified with immune signaling with high expression of inhibitory immune checkpoints, which is reported to be susceptible to immune checkpoint inhibitors (ICIs) and neoadjuvant platinum-based therapy [10], while luminal androgen receptor subtype is sensitive to androgen receptor-targeting agents [11]. In recent clinical trials (KEYNOTE-012, KEYNOTE-086, JAVELIN, IMpassion130), some TNBC patients demonstrated an encouraging response to the ICIs as single-agent immunotherapy or in combination with conventional chemotherapy [13,14,15,16,17]. Unfortunately, some patients with high expression of programmed death-ligand 1 (PD-L1) tumors still succumbed to the disease progress. In spite of current advancements or attempts at using targeted therapies for management of TNBC diseases, the high morbidity and mortality due to the development of metastases to major organs as well as drug resistance still means discovery of effective molecular targets and therapeutic strategies for TNBC are urgently needed.

Plants possess a variety of bioactive components, which have been historically applied for the treatment of numerous diseases in humans, including cancer. More than half of currently used anticancer drugs are either natural products or natural product derivatives, and several important chemotherapeutic drugs are derived from plants, such as taxanes and their analogs, camptothecin and its derivatives, vinca alkaloids vinblastine and vincristine, podophyllotoxin and its derivatives, etc. [18]. In other words, plant-derived bioactive compounds serve as a reservoir of potential chemicals for new drug development against various cancers. This review highlights and updates current plant-derived chemotherapeutic drugs for TNBC therapy and selected bioactive plant natural products covering three major types of phytocompounds, terpenoids, phenolics, and alkaloids, focusing on their bioefficacy in TNBC prevention or therapy. The most up-to-date knowledge and progress with regard to how plant-derived anticancer drugs and bioactive phytocompounds inhibit TNBC cell activity, tumor growth and metastasis, and the underlying modulatory mechanisms deregulating tumor metabolism and TME in vitro and in vivo and/or in clinical studies are summarized and discussed. A comprehensive literature search was performed in PubMed using the following specific medical subject heading (MeSH) terms. The sets of relevant keywords used in combination were: (1) “herbal medicine” OR “natural product” OR “terpenoids” OR ”polyphenols” OR “alkaloids”; (2) “triple-negative breast cancer” OR “basal type of mouse or human cell lines” OR “breast cancer”; (3) “tumor microenvironment” OR “immune” AND “therapy” OR “cytokines”; (4) “metabolism”; (5) “drug resistance”. Research studies that were validated in vivo or that discuss effects, therapeutic potentials, signaling mechanisms, or mechanisms of action were chosen for further analysis. Research studies that supported these aforementioned initial screening studies were also included for further consideration. Studies focusing on phytomedicines or phytoagents engaged in clinical trials as anti-TNBC therapies were included for further consideration according to the information provided in ClinicalTrials.gov (https://clinicaltrials.gov/, accessed on 10 December 2021). The literature search was strictly restricted to articles published within the last 11 years, from January 2010 until June 2021.

## 2. Plant-Derived Compounds Inhibit Cell Proliferation, Tumor Growth/Metastasis, and Induction of Programmed Cell Death in TNBC

### 2.1. Phenolics

Curcumin (1,7-bis-(4-hydroxy-3-methoxyphenyl)-1,6-heptadiene-3,5-dione), a multifunctional phytocompound found in turmeric root exerts antioxidant [19], anti-inflammatory [20], anticancer [21], antiaging [22], and immunomodulatory [23] effects. In TNBC cells, curcumin preferentially induced DNA damage associated with phosphorylation, increased expression, and cytoplasmic retention of BRCA1 and induction of cell apoptosis. Notably, these curcumin-induced effects were not observed in non-transformed mammary epithelial cells, suggesting that curcumin may have limited non-specific toxicity [24]. Curcumin has been reported to effectively chemosensitize breast cancer cells, independent of their receptor status, to chemotherapeutic drug 5-fluorouracil through thymidylate synthase-dependent downregulation of nuclear factor kappa-light-chain-enhancer of activated B cells (NF-κB), thereby reducing the 5-fluorouracil toxicity and drug resistance [25]. NF-κB is a transcription factor involved in the regulation of inflammatory responses and facilitating tumorigenesis and cancer progression [26]. Curcumin can suppress tumor necrosis factor (TNF)-mediated NF-κB activity and NF-κB-regulated downstream genes involved in cell proliferation (cyclin D1, and c-myc), antiapoptosis (inhibitor of apoptosis protein-1, inhibitor of apoptosis protein-2, X-linked inhibitor of apoptosis protein (XIAP), B-cell lymphoma-2 (Bcl-2), B-cell lymphoma-extra large (Bcl-xL), Bfl-1/A1, and TNF receptor-associated factor 1), metastasis and angiogenesis (cyclooxygenase-2 (COX-2), vascular endothelial growth factor (VEGF), matrix metalloproteinases-9 (MMP-9), and intercellular adhesion molecule-1) [27], resulting in inhibition of inflammatory processes and TNBC tumor growth and metastasis [28]. A combination of curcumin and arabinogalactan, a polysaccharide extracted from the wood of larch tree [29], promoted human MDA-MB-231 cell apoptosis by increasing reactive oxygen species (ROS) level, disrupting mitochondrial membranes and reduction in glutathione. The two natural product combination also inhibited the growth of mouse 4T1 TNBC cells through induction of the overexpression of p53 and reduction in Ki67 levels in vivo [30].

Resveratrol, a stilbenoid (3,5,4′-trihidroxystilbene) present in red wine, grapes, plums, berries, peanuts and pine nuts, possesses antioxidant, anti-inflammatory, and anticancer properties [31,32]. These bioactivities of resveratrol are associated with inhibition of NF-κB [33] and signal transducer and activator of transcription 3 (STAT3) [34] signaling. Sirtuin-1 is a NAD^+^-dependent histone deacetylase (HDAC) [35]. Resveratrol is a sirtuin-1 activator and can inhibit STAT3 acetylation through sirtuin-1-mediated deacetylation of key STAT3 lysine residues [36,37]. Disabling acetylation of STAT3 at K685 by HDAC antagonizes STAT3 dimerization, nuclear translocation, DNA binding, and STAT3-regulated target gene expression [38]. Although STAT3 is a well-known transcriptional activator for many genes [39], acetylated STAT3 also inhibits gene expression through interacting with DNA methyltransferase 1, resulting in an increase in CpG island methylation of certain tumor-suppressor genes [40]. Silencing of the gene encoding estrogen receptor-α (ERα) via CpG island methylation precludes the use of antiestrogen therapeutics in breast cancer patients [41]. Reduction in acetylated STAT3 by resveratrol in TNBC cells led to demethylation and re-expression of the *ERα* gene subsequently sensitizing TNBC cells to antiestrogen treatment. Resveratrol treatment sensitized TNBC cells to tamoxifen-induced cell death, and co-treatment of resveratrol and tamoxifen effectively blockaded TNBC tumor growth in a xenograft model [42]. On the other hand, resveratrol prevented dimethylbenzanthracene (DMBA)-induced mammary carcinogenesis by inhibiting 5-LOX [43] or DMBA-induced COX-2 and MMP-9 expressions in the breast tumor [44]. It is known that breast cancer stem cells (BCSCs) can promote breast tumorigenesis and progression [45]. Resveratrol inhibited cell proliferation and reduced the size and number of mammosphere formation of BCSCs isolated from MCF-7 and SUM159 (a mesenchymal TNBC cell line) cells; moreover, resveratrol effectively inhibited the growth of human SUM159 xenograft tumors in mice [46]. Resveratrol induced autophagy, as revealed by upregulation of LC3-II, Beclin1 and ATG 7, through suppression of the Wnt/β-catenin signaling pathway in BCSCs, while overexpression of β-catenin markedly reduces resveratrol-induced cytotoxicity and autophagy in BCSCs [46].

Epigallocatechin gallate (EGCG) is the component of green/black tea, grapes, cherries, apricots, and peaches. EGCG exhibits several types of bioactivities, with its anticancer effects attracting most attention [47]. EGCG exhibits significant chemopreventive effects and anticancer stem cell activity, as shown by a significant decrease in the size and number of tumors induced by DMBA in rats, significant amelioration of the oxidative stress markers, and inhibition of CD44, VEGF, Ki-67, and MMP-2 expression, together with significantly increased expression of caspase-3 [48]. EGCG decreased expression of genes promoting proliferation (*cyclin D1 (CCND1)*), migration (*RHOC, fibronectin (FN1)*), invasion (*E-cadherin (CDH1), vimentin (VIM)*), survival (*BCL-XL*), and angiogenesis (*VEGF-D*) in SUM-149 cells. EGCG was also reported to inhibit the sphere formation of BCSCs from human SUM-149 cells [49]. In an orthotopic mouse model, EGCG decreased tumor growth derived from aldehyde dehydrogenase (ALDH)-expressed BCSC-like SUM-149 cells and expression of VEGF-D, which was correlated with a significant decrease in peritumoral vessel density [49]. Furthermore, EGCG inhibited the growth of BCSCs of MDA-MB-231 and MDA-MB-436 cells and reduced the expression of *ER-*α*36* in these TNBC cells [50]. ER-α36 is a variant of ER-α overexpressed in ER-negative breast cancer cell lines, including MDA-MB-231 and MDA-MB-436 cells [51]. ER-α36 interacted with the epidermal growth factor receptor (EGFR)/Src/Shc complex and mediated estrogen-induced phosphorylation of EGFR and Src to promote malignant growth of TNBC cells in response to mitogenic estrogen stimulation [51]. ER-α36 expression is also found to correlate with ALDH1 expression, a marker of BCSCs, in clinical samples of breast cancer patients [52]. In *ER-*α*36* knocked-down MDA-MB-231 and MDA-MB-436 cells, the inhibition of the growth of BCSCs by EGCG was lost, suggesting that ER-α36 might be the target of EGCG in inhibition of growth of ER-negative BCSCs. Future preclinical and clinical evaluations of EGCG as a therapeutic option for ER-α36 positive TNBC cells were proposed [50]. Combined treatment of curcumin and EGCG reduced the BCSC-like CD44-positive cell population and mammosphere formation derived from MDA-MB-231 cells; moreover, curcumin and EGCG co-treatment synergistically inhibited STAT3 phosphorylation in MDA-MB-231 cells [53]. STAT3 signaling is known to be selectively activated in BCSCs [54], suggesting that targeting the STAT3 signaling pathway using phytochemicals is a potential direction to suppress BCSCs for treating breast cancer.

### 2.2. Terpenoids

Thymoquinone is a monoterpenoid and main ingredient in the essential oil of *Nigella sativa L.* that has been extensively utilized in the Middle East and Southeast Asia due to its multiple pharmacological properties [55]. Thymoquinone has been shown to inhibit the protein and mRNA expression of eukaryotic elongation factor 2 kinase (*eEF-2K*) (a critical factor associated with poor patient survival and prognosis) and its downstream signaling molecules, such as Src/Focal adhesion kinase (FAK) and Akt in TNBC, via induction of the tumor suppressor *miRNA-603* through suppression of NF-κB [55]. These thymoquinone bioactivities can result in a significant decrease in TNBC cell proliferation, colony formation, migration, and invasion. Furthermore, thymoquinone induced p38 phosphorylation and ROS production and inhibited protein expression of antiapoptotic factors, such as XIAP, survivin, Bcl-xL, and Bcl-2, in MDA-MB231 breast cancer cells, which can be further enhanced when combined with doxorubicin [56]. Due to the compound hydrophobicity, thermal instability, and rapid degradation features of thymoquinone, thymoquinone-loaded liposomal nanoparticles and hyaluronic acid-conjugated copolymer nanoparticles (HA-TQ-Nps) [57] were developed to enhance the efficiency of thymoquinone. Systemic in vivo liposomal thymoquinone injection significantly reduced the growth of orthotopic MDA-MB-231 tumors and inhibited the eEF-2K expression in TNBC tumors [55]. HA-TQ-Nps has been shown to inhibit cell migration and disrupt actin organization through up-regulation of *miRNA-361*, which can down-regulate Rac1 and RhoA expression, and also perturb cancer cell migration and vascularization under the influence of the autocrine effect of VEGF-A [57].

TQFL12, a synthetic derivative of thymoquinone, has more potent cytotoxic, antimetastatic, and anti-invasive bioactivities than thymoquinone by activating the AMP activated protein kinase (AMPK)/acetyl-CoA carboxylase pathway via stabilizing AMPK α subunit, a cellular energy sensor with key roles in regulating energy hemostasis and cellular metabolism [58].

Deoxyelephantopin (DET), a major germacranolide sesquiterpene lactone from the medicinal plant *Elephantopus scaber* L., has been reported to have various anti-breast cancer effects. DET showed significant antitumor growth and metastasis effects against murine estrogen receptor-expressed but highly malignant and metastatic TS/A mammary adenocarcinoma, and was superior to paclitaxel in prolonging median survival time and suppressing lung metastasis in syngeneic mice [59]. DET inhibited colony formation, cell proliferation, migration, and invasion, and induced G_2_/M arrest and apoptosis in TS/A cells [59]. DET is a calpain inhibitor and impeded metastatic mammary cell motion through deregulating actin cytoskeletal protein networks, calpain-mediated proteolysis of FAK proteins, and deactivation of Rho GTPase Rac1 to prevent lamellipodia [60]. Of note, DET-induced ROS were the upstream stimulus for the formation of centrosomal aggresomes to restrict cancer cell motility [60]. Furthermore, DET induced endoplasmic reticulum (ER) stress-mediated apoptosis by regulating protein disulfide isomerase, 78 kDa glucose-regulated protein (GRP78), thioredoxin domain-containing protein 5, caspase-12, caspase-3, and PARP proteins, and suppressed proteasomal proteolysis in breast cancer cells [61]. DETD-35, a novel semi-synthetic analog from DET, exhibited a better inhibition of migration, invasion, and motility of MDA-MB-231 cells than parental DET and significantly suppressed metastatic pulmonary foci information along with expression level of VEGF and COX-2 in the MDA-MB-231 xenograft mouse model [62]. Additionally, DETD-35 in combination with paclitaxel acted synergistically in inhibiting tumor growth and metastasis in mouse lung tissues [62]. Notably, DET and DETD-35 can differentially induce ROS-mediated apoptosis and paraptosis and autophagosome accumulation in TNBC cells through deregulating mitogen-activated protein kinase pathways and autophagosomal pathways [63].

Artemisinin is a sesquiterpene lactone isolated from Sweet Wormwood or *Artemisia annua* (called *qinghao* in Mandarin). It is a traditional medicinal plant used against malaria in the form of tea or pressed juice [64]. Recently, *A. annua*-derived compounds have gained increasing attention for their anticancer properties. Dihydroartemisinin, a principal active metabolite of artemisinin, was shown to increase early apoptosis, cell cycle arrest, and reduce tumor growth [65]. When dihydroartemisinin was delivered with docetaxel in pH-sensitive nanoparticle delivery form [66] or in disulfide-linked nanoparticle delivery form [65], these dihydroartemisinin-docetaxel nanoconjugates showed stronger effects on inhibiting tumor growth and metastasis and promoting survival of 4T1 tumor-bearing mice than monotherapy of either drug through improvement of drug uptake by cells and pharmacokinetics/pharmacodynamics. Furthermore, artemisinin-loaded biotin-poly(ethylene glycol) and poly(ξ-caprolactone) polymers [67] or chitosan magnetic nanoparticles [68] were also shown to improve the pharmacokinetics and antitumor efficacy in the 4T1 mouse model. Artesunate, a semi-synthetic compound derived from artemisinin, has recently revealed remarkable antitumor activity as a novel candidate for cancer chemotherapy. Artesunate can induce caspase-9-dependent apoptosis through down-regulating the expression of Bcl-2 and heat shock protein 70 in 4T1 cells [69]. Recently, a study showed that dimeric artesunate-phosphatidylcholine conjugate liposomes (dARTPC), when encapsulated with irinotecan, exhibited more significant antitumor effects than monotherapy of dARTPC or irinotecan in a 4T1 tumor model [70]. However, unexpectedly, other studies reported that artesunate can potentially induce drug resistance toward doxorubicin or itself in HT29 through calcium-dependent activation of HIF-1α and P-glycoprotein (P-gp) overexpression and MDA-MB-231 cells through activation of NF-κB and activator protein 1 [71,72].

Paclitaxel (Taxol^®^) is a natural tricyclic diterpenoid with a taxane ring identified in *Taxus brevifolia* [73]. Taxol was first approved by the U.S. FDA in 1993, which marked the great entrance of terpenoids into the chemotherapeutic arena. Paclitaxel is an important frontline chemotherapy drug, especially for advanced metastatic cancers [74]. Unlike most antitumor drugs that damage DNA or RNA, the main mechanism of paclitaxel was to promote cellular death through causing mitotic arrest via binding to tubulin and inhibiting the disassembly of microtubules [75]. In addition, paclitaxel can trigger mitochondrial apoptosis via release of Ca^2+^ and mitochondrial damage, leading to ROS production and Bcl-2 associated X (BAX) expression and reduction in antiapoptotic protein Bcl-2 [75].

Triptolide, a natural diterpenoid epoxide isolated from the *Tripterygium wilfordii*, possesses anti-inflammatory and antitumor activities involving multiple molecular targets and signaling pathways. Triptolide significantly suppressed MDA-MB-231 cell viability and clonogenic ability via inhibiting *High Mobility Group Box 1 (HMGB1)* mRNA expression and its associated factors, e.g., Toll-like receptor 4 (TLR4) and phosphorylated form (p) of NF-κB p65 and inhibited MDA-MB-231 tumor growth [76]. Of note, there is one ongoing clinical study to evaluate the safety, pharmacokinetic, and pharmacodynamics of Minnelide Capsules (triptolide) in patients with advanced solid tumors when given alone or in combination with paclitaxel (NCT03129139).

### 2.3. Alkaloids

Camptothecin is a quinolone alkaloid found in the bark/stem of *Camptotheca acuminate*, *Chonemorpha fragrans*, and *Nothapodytes nimmoniana*. It is a topoisomerase I (TOP1) inhibitor trapping TOP1 cleavage complexes, which resulted in dsDNA breaks during replication. Due to poor water solubility and non-specific killing effect, camptothecin has been developed and loaded into nanoparticles carrying the EGFR-targeting antibody cetuximab to generate a soluble and directed nanoscale delivery vehicle [77]. In mice bearing bone metastatic MDA-MB-231 cells, treatment with camptothecin-loaded nanoparticles inhibited primary tumor growth, decreased lung, liver, and bone metastases, and increased mouse survival rate [77]. These active targeting strategies effectively circumvent inherent pharmacological limitations of camptothecin. CRLX101, a nanoparticle–drug conjugate containing camptothecin, was also designed to improve camptothecin solubility, stabilize its lactone ring, and reduce systemic toxicity. Bevacizumab, an anti-VEGF antibody to reduce tumor angiogenesis, was found to induce tumor hypoxia and upregulation of hypoxia-inducible factor-1α (HIF1α), resulting in hypoxia-induced resistance to bevacizumab in tumors [78]. CRLX101 can suppress tumor hypoxia and HIF1α expression, thus counteracting undesirable effects caused by bevacizumab. Furthermore, CRLX101 combined with bevacizumab led to complete tumor regressions, reduced lung metastasis, and greatly extended survival of mice bearing a highly aggressive variant of MDA-MB-231 cells or a patient-derived xenograft [79]. On the other hand, irinotecan, an analogue made from camptothecin, exhibited a much better water solubility than camptothecin and has been approved for medical use to treat colon cancer patients in the United States since 1996 [80]. Irinotecan in combination with iniparib, an inhibitor of PARP, was used to treat TNBC patients with brain metastases, showing modest benefits [81].

Sacituzumab govitecan is a conjugate of the humanized antibody linked with SN-38 (7-ethyl-10-hydroxy-camptothecin), the active metabolite of irinotecan. The antibody–drug conjugate binds to trophoblast cell-surface antigen 2 (Trop-2)-positive cancer cells, delivering the chemotherapy drug SN-38 directly to Trop-2-positive cells. Sacituzumab govitecan significantly prolonged the progression-free and overall survival as compared to single-agent chemotherapy in patients with metastatic TNBC [82,83], especially in TNBC patients expressing high/medium levels of Trop-2 [84]. Combining sacituzumab govitecan with PARP inhibitors in vitro resulted in synergistic growth inhibition and increased dsDNA breaks, as revealed by γ-H2A histone family member X (γ-H2AX), in TNBC cells, regardless of *BRCA1/2* status. γ-H2AX is the first step in recruiting DNA repair proteins and is a biomarker for dsDNA breaks [85]. The combination therapy triggers more significant antitumor effects against tumor growth than those achieved with monotherapy in xenograft mice bearing *BRCA1/2*-wildtype or mutated TNBC tumors [86].

Etoposide (VP-16, epipodophyllotoxin) is a semisynthetic derivative of podophyllotoxin, an alkaloid from the mandrake plant *Podophyllum peltatum*. It is a topoisomerase II inhibitor that has been approved for clinical use as a form of chemotherapy for cancers in the USA since 1983 [87]. Oral etoposide monotherapy was effective in Chinese [88,89] or in Italian [90] women with heavily pretreated metastatic breast cancer. Combined etoposide treatment modalities were also tested in preclinical TNBC animal models. TMU-35435 (*N*-hydroxy-6-(5-methyl-4-acridinecarbamoyl) hexanamide), a HDAC, can inhibit DNA repair proteins which enhances etoposide-induced DNA damage by inhibiting the DNA repair pathway (non-homologous end joining). The combined treatment with TMU-35435 and etoposide effectively reduced TNBC tumor growth in vivo. The antitumor effects of combination treatment may be related to the induction of apoptosis, autophagy and DNA damage as revealed by increased expression of caspase-3, LC-3, and γ-H2AX in 4T1 tumor sections [91]. TNF-related apoptosis-inducing ligand (TRAIL), a member of the TNF family of ligands, triggers apoptosis selectively in cancer cells through interaction with the death receptors, DR4 and DR5. Although TRAIL can selectively induce apoptosis in cancer cells, the development of resistance is a potential limitation of TRAIL treatment [92]. Etoposide was found to modulate the TRAIL-DR5 axis. Etoposide enhanced DR5 expression in TNBC cells and induced a higher degree of apoptosis in the TRAIL pre-treated cells, suggesting synergistic effects of TRAIL and etoposide against TNBC cells. In the MDA-MB-231 xenograft tumors, the expression of DR5, PARP, caspases, and p53 proteins was increased in mice co-treated with TRAIL and etoposide [93].

Berberine, an isoquinoline alkaloid, can be found in *Coptis* and *Phellodendron* plants. Berberine exerts chemopreventive effects against DMBA-induced mammary carcinogenesis through downregulating NF-κB and proliferating cell nuclear antigen (PCNA) expression in breast tumors. Furthermore, berberine decreased the secretion of proinflammatory cytokines and the expression of P2X purinoceptor 7 (P2X7), interleukin (IL)-18, and NLR Family Pyrin Domain Containing 3 (NLRP3) in MDA-MB-231 cells [94]. Berberine inhibited P2X7-mediated NLRP3 inflammasome activation in human TNBC cells, which might provide therapeutic relevance for clinical use since targeting the NLRP3 inflammasome pathway, is suggested to be a potential strategy for cancer immunotherapy [95,96]. Vasodilator-stimulated phosphoprotein (VASP) is overexpressed in high-motility breast cancer cells, including basal-like TNBC and HER2-positive subtypes. VASP can promote actin filament elongation and induce cell proliferation, adhesion, and migration [97]. Berberine could bind directly to VASP and alter the secondary structure of VASP, resulting in inhibiting actin polymerization in breast cancer cells. Moreover, a VASP-blocking mechanism may account for the observations that berberine inhibits proliferation and migration of MDA-MB-231 cells as well as tumor growth in MDA-MB-231-bearing mice [98]. The combination of berberine and recombinant TRAIL activated caspase-3 and PARP in TRAIL-resistant MDA-MB-468 cells in vitro [99]. In the 4T1 TNBC model, berberine treatment enhanced the efficacy of anti-DR5 antibody therapy against tumor growth and lung metastasis. Thus, berberine may behave as an adjuvant to overcome the resistance of TNBC cells to TRAIL/DR5-mediated therapy [99].

### 2.4. Other Phytocompounds/Extracts

Sulforaphene (4-methylsufinyl-3-butenyl isothiocyanate), a member of the isothiocyanates, is derived from radish seeds. Sulforaphane repressed expression of cyclin B1 and Cdc2/p-Cdc2 and induced G_2_/M phase arrest and apoptosis in TNBC cells; sulforaphane inhibited tumor growth mediated by activation of tumor suppressor Egr1 in MDA-MB-453 xenograft mouse models [100]. In MDA-MB-231 tumor model, sulforaphane suppressed the malignant proliferation and mammosphere formation of BCSCs in TNBC via Cripto-mediated pathway and decreased the expression of stem-related embryonic oncogenes *CRIPTO-1/TDGF1*, *CRIPTO-3/TDGF1P3* and various stem cell markers including *aldehyde dehydrogenase 1A1 (ALDH1A1)*, *Wingless-related integration site (WNT3)*, and *Notch receptor 4* (*NOTCH 4)* [101]. Furthermore, sulforaphane inhibited MDA-MB-231 cell growth and induced autophagy by down-regulating expression of HDAC6, which resulted in increased membrane translocation and acetylation modification of PTEN [102]. Sulforaphane and doxorubicin combination exhibited a synergistic inhibition of MDA-MB-231 xenograft growth in nude mice, with greater inhibitory effect than monotherapy [102]. Sulforaphane was also shown to prevent expansion of and to eliminate taxane-induced ALDH^+^ BCSCs and dramatically reduced mammosphere formation via inhibition of NF-κB p65 subunit translocation and downregulation of p52 [103]. The combination of docetaxel and sulforaphane exhibited a greater reduction in primary tumor volume and significantly reduced secondary tumor formation [103].

A gallotannin-rich fraction derived from *Caesalpinia spinosa (P2Et)* was characterized by Fiorentino’s group, which contained 13 gallotannins characterized by HPLC/MS [104,105]. P2Et was proved to decrease clonogenic capacity and induce apoptosis with mitochondrial membrane potential loss, phosphatidylserine externalization, caspase-3 activation, and DNA fragmentation in mouse 4T1 cells and to decrease primary tumor growth and metastasis in 4T1 mouse models [104]. More importantly, P2Et extract was found to be cytotoxic and demonstrated drug efflux reversion and antioxidant activity against cancer cells exhibiting multidrug resistance mechanisms [105]. After further analyzing the bioactivity of sub-fractions of P2Et with different polarities, P2Et-mediated drug-efflux activity was shown to be governed by hydrophobic compounds, which could be possibly due to alkyl gallates [106]. The features of P2Et on modulation of drug efflux may contribute to the synergistic effect of P2Et with doxorubicin to inhibit tumor growth and prolong the survival time of mice in TS/A tumor model [105]. P2Et extract alone was able to decrease MDA-MB-468 tumor growth in vivo, and the viability of mammospheres derived from breast cancer patients was suppressed by P2Et extract alone or combined with doxorubicin [107]. A clinical trial has recently been initiated to evaluate the safety and efficacy of the P2Et extract in patients with breast cancer (NCT05007444).

**Figure 1:**
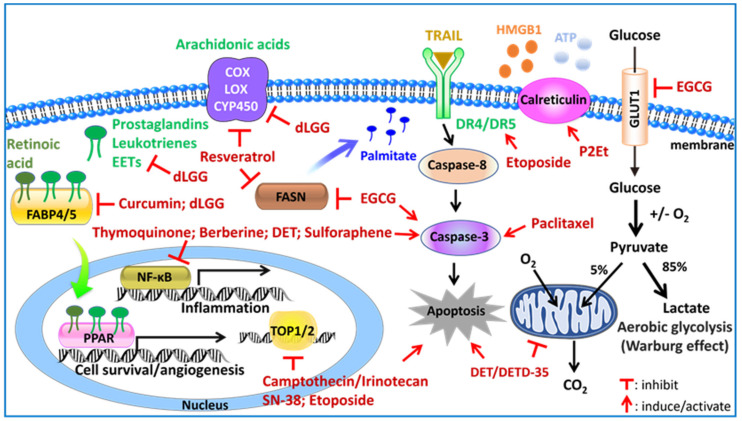
A summary of the effects and mechanisms of selected phytocompounds/extracts on the regulation of TNBC cell growth, apoptosis, and metabolism. EGCG decreases GLUT1 expression, glucose uptake, and lactic acid level and suppresses glycolytic enzymes involved in the Warburg effect. EGCG also inhibits FASN and FASN-mediated drug resistance through the overproduction of palmitate. By down-regulating FASN expression, resveratrol reduces the cell survival and mammosphere formation of BCSCs. Arachidonic acids can be metabolized by COXs, LOXs, and CYP450 epoxygenases to produce prostaglandins, leukotrienes, and EETs, respectively. Resveratrol prevents DMBA-induced mammary carcinogenesis by blocking 5-LOX or by suppressing the DMBA-induced COX-2 and MMP-9 expression in the breast tumor. Nuclear translocation of FABP4 and FABP5 mediated by lipid ligands, such as EETs, upregulates nuclear expression of PPAR and the transcription of PPAR-regulated genes. By suppressing the expression levels of FABP5 and PPARβ/δ, curcumin prevents the delivery of retinoic acid to PPARβ/δ and suppresses retinoic acid-induced PPARβ/δ target genes. A phytogalactolipid, dLGG, effectively attenuates TNBC recurrence and lung metastasis through downregulation of FABP4, FABP5, PPARγ, and EETs. Camptothecin, irinotecan, SN-38, and etoposides are TPO1/2 inhibitors. Etoposide enhances DR5 expression, promoting TRAIL-induced apoptosis. Thymoquinone, berberine, DET, and sulforaphane inhibit NF-κB activation, showing anti-inflammatory activities. Paclitaxel and other phytocompounds increase caspase-3 activity. P2Et can trigger immunogenic cell death, inducing the release of HMGB1 and ATP and expression of calreticulin.

Integrated signaling molecule/pathways, which are regulated by the discussed bioactive phytocompounds for their anti-TNBC cell or tumor growth activities, are summarized in Figure 1.

## 3. Plant-Derived Compounds Reprogram Cellular Metabolisms and Associated Proteins and Signaling Pathways in Drug Sensitive/Resistant TNBC

Metabolism deregulation is considered to be a hallmark of cancer, as cancer cells proliferate rapidly and thus need more energy to support their growth [108]. Metabolic changes drive cancer cell growth and proliferation, as well as their ability to develop drug resistance [109]. Meanwhile, tumor-infiltrating immune cells in the tumor microenvironment (TME) may also have a metabolism-altering function. For example, the enhanced metabolism of L-arginine in myeloid cells decreases the response of lymphocytes to tumor antigens, resulting in the failure of clearance of cancer cells and permission for intensive tumor growth [110]. Therefore, the metabolic features of cancer and surrounding cells within the TME can permit or even facilitate cancer cell survival, progress, and metastasis [111].

Unlike normal cells, cancer cells rely primarily on glycolysis to generate energy, even in an aerobic environment. This phenomenon, termed aerobic glycolysis or the Warburg effect, allows tumor cells to generate a high rate of glycolytic intermediates, which become substrates for several biosynthetic pathways crucial for cell proliferation and cancer progression [112]. An increased aerobic glycolysis rate is a common metabolic feature of many cancer cells, and targeting the Warburg effect has been under investigation as a therapeutic focus to improve anticancer therapy [113]. Among breast cancers, TNBC cells have increased glycolysis and concomitant decreased mitochondrial respiration as compared to other breast cancer subtypes, suggesting that TNBC cells are highly sensitive to glycolytic inhibition [114]. Glucose transporter 1 (GLUT1) is responsible for the uptake of glucose from blood into cancer cells and is overexpressed in breast cancer cells [115,116]. Elevated expression of GLUT1 in the tumor section is also observed in basal-like TNBC patients [117], suggesting an important role for GLUT1 in regulating TNBC cell metabolism. Thus, phytoagents that can regulate glucose metabolism in TNBC cells are of particular interest. EGCG has been found to decrease the expression of HIF1α and GLUT1, both of which are critical players in the modulation of glycolysis [118,119,120]. EGCG treatment inhibited 4T1 cells from uptake of glucose and production of lactic acid, resulting in a reduction in ATP levels in the treated cells. EGCG also suppressed the activities and levels of the glycolytic enzymes, such as hexokinase (HK), phosphofructokinase (PFK), lactic dehydrogenase A (LDHA), and pyruvate kinase isozyme type 2 (PKM2) in 4T1 cells. In a syngeneic animal model, EGCG reduced 4T1 tumor growth along with decrease in tumor glucose and lactic acid levels and inhibited tumor VEGF expression [121]. As compared to the luminal type of breast cancer, TNBC cells and immunohistochemistry (IHC) staining of tumor tissue microarrays from TNBC patients exhibited higher levels of LDHA expression [122]. Moreover, the increased expression and activity of LDHA were detected in taxol-resistant cells as compared to their parental MDA-MB-435 cells. Targeting LDHA by siRNA knockdown or by oxamate, an analog of pyruvate, can resensitize taxol-resistant TNBC cells to taxol treatment in vitro [123].

Under normal circumstances, fatty acid synthesis mainly occurs in lipogenic tissues, especially adipose tissue. However, fatty acid synthesis is enhanced in tumor cells to produce membrane phospholipids and signaling molecules for tumor cell proliferation [124]. Fatty acid synthase (FASN) is required for de novo synthesis of fatty acids and is correlated with poor prognosis in breast cancer patients [124]. Moreover, overexpression of FASN and fatty acid-binding proteins (such as FABP4, FABP5) may promote multidrug resistance in breast tumor cells [125]. Reducing FASN expression was observed to increase drug sensitivity in breast cancer cell lines MCF-7 and MDA-MB-468 [126]. FASN-mediated drug resistance is likely due to a decrease in drug-induced apoptosis from the overproduction of palmitic acid by FASN [126]. Overexpression of FASN may induce drug resistance by (i) altering the membrane lipid composition, thus decreasing the influx of chemotherapeutic drugs, or (ii) inhibition of apoptosis [127]. Thus, FASN may be an ideal target for chemosensitization in breast cancer chemotherapy. Remarkably, FASN inhibition has minimal effect on non-malignant cells, because FASN has a low or absent expression in normal tissues but is overexpressed and hyperactivated in many carcinomas such as breast cancer [124]. In addition, FASN is found to be overexpressed in TNBC patients [128], and the TNBC preclinical models benefit from FASN inhibition by EGCG [129]. A combination of EGCG with cetuximab (anti-EGFR antibody) displayed strong antitumor activity against the sensitive and chemoresistant TNBC xenografts, without signs of toxicity [129]. By down-regulating FASN expression and lipid synthesis, resveratrol reduced cell survival and mammosphere formation and induced apoptosis in CD24^−^/CD44^+^/epithelial-specific antigen-positive BCSCs derived from MDA-MB-231 cells. Resveratrol also suppressed the growth of BCSC-like cells in vivo and downregulated tumor FASN expression [130].

Fatty-acid binding proteins (FABPs) are a class of intracellular lipid-binding proteins that transport hydrophobic lipids throughout cellular compartments, including to the peroxisomes, mitochondria, ER, and nucleus [131]. FABP delivery of lipids to the nucleus facilitates the activation of PPARs and the transcription of PPAR-regulated genes [131]. Among the ten FABP isoforms in humans, FABP4 and FABP5 likely play critical roles in mammary tumor development [132]. FABP5 facilitates the transfer of lipid ligands from the cytoplasm to PPARβ/δ, which helps transcribe PPARβ/δ target genes that are directly involved in proliferative responses and cell survival, promoting cell growth and protection against apoptosis in breast and prostate cancers [133,134,135,136]. PPARβ/δ has been implicated in the growth of human cancers, including lung carcinoma, breast cancer, and colon cancer [137]. In TNBC patients, elevated levels of FABP5 were correlated with tumor grade and poor prognosis [138], and higher amounts of tumor FABP4 expression were associated with significantly shorter disease-free survival and overall survival [139,140]. By suppressing the expression levels of FABP5 and PPARβ/δ in TNBC cell lines, curcumin prevented the delivery of retinoic acid to PPARβ/δ and suppressed retinoic acid-induced PPARβ/δ target genes. Therefore, inhibition of the FABP5/PPARβ/δ pathway by curcumin sensitized retinoic acid resistant TNBC cells to retinoic acid mediated growth suppression [141].

Arachidonic acid metabolic pathways play important roles in the carcinogenesis and metastasis of TNBC tumors. Arachidonic acids can be metabolized by cyclooxygenases (COXs), lipoxygenases (LOXs), and cytochrome P450 (CYP) epoxygenases to produce eicosanoids, such as prostaglandins, leukotrienes, and epoxyeicosatrienoic acids (EETs), respectively [142]. Recently, Apaya et al. identified higher levels of CYP epoxygenases and EETs in the human mammary tumors compared to their adjacent normal tissues in a group of specified TNBC patients [143], indicating that arachidonic acid-derived EETs are important drivers of metastasis in TNBC tumors. Meanwhile, CYP epoxygenase overexpression predicts metastasis risk and is associated with shorter survival in TNBC patients, and EETs can promote migration, invasion, and metastasis phenotype in mesenchymal-like TNBC cells [143]. Nuclear translocation of FABP4 and FABP5 mediated by lipid ligands, such as EETs, upregulates nuclear expression of PPARγ and the transcription of PPAR-regulated genes, including FABP4 and VEGF [144,145]. A phytogalactolipid, 1,2-di-*O*-α-linolenoyl-3-*O*-β-galactopyranosyl-*sn*-glycerol (dLGG), effectively attenuated TNBC recurrence and lung metastasis through downregulation of FABP4, FABP5, PPARγ, and EETs, resulting in inhibition of the EET/FABP-mediated signaling axes in TNBC relapse and metastasis [140].

Although chemotherapy is the main therapeutic option for TNBC patients, acquired drug resistance may develop after long-term treatment. The tumor metabolism and tumor microenvironment with surrounding immune cells may give rise to a new subset of cancer cells that are resistant to drug treatment and show continuous growth and metastasis [146,147]. Phytochemicals with the potential to change tumor metabolism or the tumor microenvironment may provide opportunities to overcome chemoresistance. The occurrence of chemoresistance is a major factor driving the failure of paclitaxel treatment and breast cancer relapse. Paclitaxel resistance pathways identified to be involved in major regulatory proteins/RNAs, such as RNF8/Twist/ROR1, TLR, ErbB3/ErbB2, BRCA1- IRIS, MENA, LIN9, micro-RNAs (miRNAs), FoxM1, and IRAK1, have expanded the complexity of resistance mechanisms [148]. Furthermore, the aberrant levels of multi-antennary branching structures and their target glycosyltransferases, *N*-glycosylation and glycoproteins, were associated with paclitaxel resistance [149]. In addition, chemoresistance of paclitaxel has also been reported to be associated with autophagy and dysregulation of the TLR4 and MyD88-dependent pathways [150,151,152]. Recent studies exploring the mechanism of development of paclitaxel resistance have led to the unveiling of a range of novel therapeutic targets. For example, EGCG can sensitize TNBC cells to paclitaxel-induced cytotoxicity, overcome paclitaxel-induced GRP78 expression, and potentiate paclitaxel-induced JNK phosphorylation in 4T1 cells both in vitro and in vivo [153]. The sequential release of EGCG followed by paclitaxel from the core/shell PLGA-casein nanoparticle sensitized paclitaxel resistant MDA-MB-231 cells to paclitaxel, induced their apoptosis, inhibited NF-κB activation, and downregulated the key genes associated with angiogenesis, tumor metastasis, and survival [154]. Curcumin has the potential to reverse mechanism involved in acquisition of drug resistance through downregulation of P-gp expression that might provide a good rationale for combination treatment of curcumin and docetaxel [155].

## 4. Plant-Derived Compounds Educate the Tumor Microenvironment and Immune Checkpoints Activity-Associated Signaling Molecules and Pathways

The tumor microenvironment (TME) contains cancer-associated fibroblasts, immune cells, adipocytes, extracellular matrix, blood and lymphatic vessels, and mesenchymal cells [156]. The interaction among these components within the TME might regulate immunosurveillance exerted by infiltrating immune cells to eliminate the transformed cells before tumors become clinically detectable, while immunoevasion happens when tumor cells become less immunogenic and cannot be killed by the surrounding immune cells. The immunosuppressive mechanisms established in the TME may make cancer cell clones invulnerable to immune recognition and killing and turn immune cells into tumor-supporting cells, hence allowing for cancer growth and dissemination [157,158]. Therefore, therapeutic intervention that reprograms the TME from favoring immunoevasion, instead directing it to immunosurveillance, may provide an opportunity to prevent tumors from further growth or metastasis and even totally eradicate cancer cells.

Immunosurveillance is an important front-line defense to recognize and eliminate neoplastic cells; however, this can be altered by more-virulent neoplastic cells through immunoediting and creation of immunosuppressive TME [159]. Immune cell profiles and immune score in breast TME are well characterized and are associated with cancer progression and survival [160,161,162]. In TME of TNBC, the infiltration of CD8^hi^ T cells were positively correlated with PD-L1 expression [163]. A higher rate of tumor immune infiltration is associated with a better outcome and reduced risk of recurrence in TNBC. Infiltration of T cells is associated with improved prognosis in TNBC [161], whereas γδ T cell infiltration is positively correlated with advanced tumor stages [164]. Immunosuppressor myeloid cells including tumor-associated macrophages (TAM) and myeloid-derived suppressor cells (MDSCs) play a prominent role in immune suppression and protumorigensis in the TME. They are very important partners of cancer cells in the process of evading antitumor immunity via suppression of the mechanisms for activation of adaptive immune responses, leading to tumor cell growth, invasion, and metastasis [165,166,167]. Tumor cells can secrete colony stimulating factor-1, chemokine (C-C motif) ligand 2 (CCL2), chemokine (C-C motif) receptor 5 (CCR5), chemokine (C-X-C motif) ligand 1 (CXCL1), and so on, to recruit immunosuppressive myeloid cells toward the TME. MDSCs can inhibit the proliferation of CD4^+^ and CD8^+^ T-lymphocytes and block the activation of NK cells, negatively affecting the maturation and function of antigen-presenting dendritic cells (DCs) [166,167]. TAM can upregulate annexin-2 and promote proliferation, angiogenesis, F-actin polymerization, and the PI3k/AKT/GSK3β/Snail signaling pathway through CCL-18-Nir-1-axis signaling [168,169]. Neutrophils are efficient producers of ROS and Arginase 1 (ARG1), which can promote tumor growth. Tumor-associated neutrophils can induce ARG1-dependent immunosuppression through concomitant exocytosis of gelatinase and azurophil granules. Absolute lymphocyte counts and the neutrophil-to-lymphocyte ratio can be used to identify high risk TNBC patients who may benefit from clinical trials rather than standard of care therapy [170]. These tumor-associated immunosuppressor cells will also activate regulatory lymphocyte development, regulatory B and T cells (Bregs and Tregs) that regularly infiltrate into the TME causing immunosuppression by releasing cytokines and upholding tumor progression [171].

**Figure 2:**
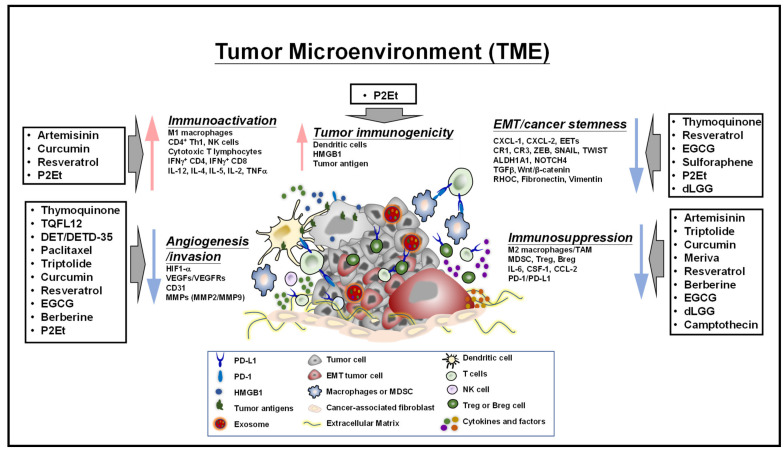
The immunomodulatory effects of phytocompounds in the tumor microenvironment. The tumor microenvironment is composed of heterogeneous tumor cells, cancer-associated fibroblasts, and various immune cells, including antigen-presenting cells-DCs and macrophages, immuno-suppressive cells (TAMs, MDSCs, regulatory T and B lymphocytes), and effector cells (NK, Th1, and cytotoxic T lymphocytes). TME creates a niche for the mutual interactions between malignant tumor cells and neighboring cells, resulting in cellular and molecular changes and fostering critical events that control the balance between immunosurveillance and immune escape, fine-tunes tumor immunogenicity, and supports EMT, cancer stemness, invasion, metastasis, and angiogenesis. This review provides several exemplar phytocompounds that confer high therapeutic potentials targeting key autocrine/paracrine cytokines, lipid mediators, and/or tumor immune infiltrates involved in antitumor immunities, tumor immunogenicity, and tumor progression.

The pathways regulating TME are critical for cellular mechanisms such as proliferation, differentiation, and survival. TME-mediated growth factor receptors, chemokines (CCL2, CCL5), and cytokines (IL-1β, IL-6, TNFα) are associated with aggressiveness of cancers and poor prognosis of patients [172]. TME consists of various proangiogenic factors (VEGF, fibroblast growth factor, and platelet-derived growth factor) and chemokines that activate neovascularization essential for the survival of the hypoxic condition [173]. Exosomes are nanovesicles that are enriched with lipids, proteins, nucleic acids, and miRNAs during biogenesis, mediate horizontal transfer from donor to recipient cells [174], and have recently been recognized as promising mediators of tumor–host interactions, communicators of TME, and vital components in the tumorigenic microenvironment [175]. Dormant blood vessels, pericytes, tumor-associated adipocytes, and other stromal cells are key players in providing fuel and growth factors, and suppressing drug responses, as well as supporting neoangiogenesis and epithelial mesenchymal transition (EMT) and hypoxia status [176,177,178]. Phytocompounds modulate the TME through targeting key autocrine/paracrine cytokines, lipid mediators, and tumor immune infiltrates involved in antitumor immunities, tumor immunogenicity, and tumor progression (EMT, cancer stemness, invasion, metastasis, and angiogenesis) are depicted in Figure 2.

### 4.1. Regulation of Tumor-Infiltrating Cells, Tumor Cell-Immune Cell Interactions, and Associated Signaling Molecules in the TME

Through modulation of tumor-infiltrating cells (TILs) and cytokine secretions in the TME, curcumin may improve the efficacy of combination therapy. Curcumin improved the efficacy of attenuated Listeria-Mage-b therapeutic vaccine against metastases in the TNBC model through reversal of tumor-induced immune suppression. Mage-b is homologous to Mage-a [179], and human homologue Mage-a is expressed in 26% of the TNBC patients [180]. Listeria-Mage-b vaccination is effective against metastatic breast cancer in 4T1-bearing mice in a preventive setting but less effective in a therapeutic setting [181]. When curcumin was orally administered before tumor development in combination with Listeria-Mage-b therapeutic vaccine, the production of IL-6 was significantly decreased, and IL-12 was increased by MDSCs in correlation with improved CD4^+^ T and CD8^+^ T cell responses in blood [182]. The metformin and curcumin combination exhibited the best effects against tumor proliferation and growth as compared to either drug treatment alone. This combined treatment significantly reduced VEGF expression, triggered Th2 immune response with increased serum IL-4 and showed no toxicity [183].

Curcumin monotherapy upregulated miR181b and downregulated miR181b targets, CXCL-1 and CXCL-2, in cells isolated from primary human breast cancers and MDA-MB-231 cells. Through inhibition of CXCL-1 and CXCL-2, curcumin diminished the formation of breast cancer lung metastases [184]. Meriva is a lecithin delivery system of curcumin with improved bioavailability. Cryoablation involves killing tumor cells through freezing and thawing, resulting in recruitment of tumor-specific T cells. Meriva administration after cryoablation stimulates CD8^+^ T cells to multiple tumor-associated antigens such as Mage-b and survivin (both expressed in 4T1 tumors), probably through the reduction in IL-6 in the TME. The combined treatment resulted in a nearly complete reduction in 4T1 primary tumors and lung metastases [185]. Resveratrol attenuated tumor growth and lung metastasis of 4T1-bearing mice by preventing the generation and function of tumor-evoked regulatory B cells (Bregs) and the expression of transforming growth factor beta (TGF-β). As a result, inhibition of Bregs by resveratrol suppresses the conversion of Treg cells through the reduction in TGF-β production by Bregs [186].

Clinical outcomes can be improved, or side effects can be minimized, if the overall doses of chemotherapy are reduced without compromising the therapeutic efficacy in a combinational therapeutic approach. Treatment of camptothecin (1.2 mg/kg/dose) plus doxorubicin (2 mg/kg/dose) at low doses shows synergistic effects against 4T1 and MDA-MB-231 tumor growth in an orthotopic model and significantly prolongs mouse survival. In 4T1-bearing mice, the combined treatment inhibits M2 TAM and induces comparably higher amounts of CD8^+^ T cell infiltration into the tumor site, which might account for the better anticancer efficacy in the combination therapy [187]. Co-loaded liposome of berberine and doxorubicin can decrease the myocardial rupture toxicity caused by doxorubicin but more significantly inhibit tumor growth in 4T1-bearing mice as compared to single-drug loaded liposomes [188]. Artemisinin has been reported to impede tumor growth and extend survival of 4T1-tumor bearing mice by quenching immunosuppression from MDSCs and Tregs and activating T cell responses [189]. In the study, artemisinin was shown to significantly decrease *TGF-β* mRNA levels and populations of MDSCs and Treg cells within the tumor, while increasing *TNF-α* mRNA levels, CD4^+^ interferon gamma-expressed (IFN-γ^+^) T cells, and cytotoxic T lymphocytes [189]. Triptolide selectively inhibited macrophage differentiation toward the M2 phenotype through downregulation of CD206, arginase 1, and CD204, secretion of anti-inflammatory cytokines, and abolished M2 macrophage-mediated tumor progression [190]. P2Et can decrease the number of peripheral blood leukocytes (leukemoid reaction) and IL-6 serum levels, which are events associated with a poor prognosis [104]. In the meantime, P2Et fraction can induce expression of immunogenic cell death markers, such as calreticulin, HMGB1 translocation from nuclei to cytoplasm, and ATP secretion [191]. When vaccinated with P2Et-pretreated 4T1 cells, the primary tumor growth was improved by yielding IL-2, TNFα, IL-4, IL-5, and IFNγ-producing CD4^+^ and CD8^+^ T lymphocytes [191]. Prophylactic treatment of healthy mice with P2Et increased the numbers of CD4^+^ and CD8^+^ activated T cells, NK cells, and DCs; however, Treg cells, MDSC, and IL-6 were also increased, which may result in failure to establish a protective effect against the control of tumor growth and metastasis in transplantable models of breast cancer [192]. Furthermore, P2Et or PD-L1 alone showed significant antitumor effects and granulocyte reduction [193]. P2Et plus PD-L1 therapy showed no clear additive effect on tumor growth and other immune cell subsets in the 4T1 tumor model [193] but had a synergistic antitumor effect in B16 melanoma models along with increased leukocytes, lymphocytes, monocytes, and granulocytes [193]. These observations indicate that P2Et could be a potential antitumor candidate as monotherapy or immune adjuvant for cancer patients by enabling the activation of the immune responses.

As proliferation and metastasis of cancer cells rely on oxygen and nutrient supply from nearby blood vessels, phytochemicals with the ability to target angiogenesis are of particular interest. In human MDA-MB-231 cell-bearing xenograft mice, curcumin treatment decreases the expression of VEGFR2/3 and micro-vessel density in the tumor, suggesting that curcumin could suppress tumor growth through inhibition of angiogenesis in the TME [194]. Calcitriol is a multitarget anticancer hormone. The combined treatment of calcitriol and curcumin synergistically reduced the tumor growth and micro-vessel density in the TNBC (MBCDF-T cell)-bearing nude mice [195]. Thymoquinone-loaded, hyaluronic acid-conjugated copolymer nanoparticles can affect cancer cell migration under the influence of the autocrine effect of VEGF-A and perturb tumor-induced vascularization by reducing the secretion of VEGF-A [57]. On the other hand, triptolide could significantly decrease the expression of VEGF-A in Hs578T and MDA-MB-231 breast cancer cells. Triptolide also reduced tube formation of human umbilical vein endothelial cells and angiogenesis through inhibiting the ERK1/2-HIF1-α-VEGFA axis [196]; triptolide decreased VEGFA, cluster of differentiation (CD) 31 in MDA-MB-231 tumor.

### 4.2. Regulation of Immune Checkpoint Expression and Activity

Cancer cells can escape immunosurveillance through immune suppression by expressing ligands for co-inhibitory receptors such as programmed death-1 (PD-1) or cytotoxic T-lymphocyte protein 4 (CTLA-4). Immune checkpoint inhibitors (ICIs), such as antibodies against CTLA-4, PD-1, and its ligand PD-L1, can restore immune cell activation, which aids anticancer immune responses for cancer immunotherapy [197,198]. Breast cancer cells with epithelial markers express high levels of MHC-I, low levels of PD-L1, and contain CD8^+^ T cells and M1 macrophages within the tumor stroma, which might contribute to a better response to anti-CTLA-4 treatment. In contrast, those with mesenchymal markers express low levels of MHC-I and high levels of PD-L1 and contain exhausted CD8^+^ T cells and M2 macrophages within the tumor stroma, which might result in immunoevasion [199]. Thus, phytoagents may be able to strengthen anticancer responses through modulation of immune checkpoint expression.

Suppression of TNBC lung metastasis by resveratrol may be through elevating local antitumor immunity, as revealed by an increase in the levels of type 1 cytokines, including IFN-γ and IL-2 in the lung and infiltration of CD4^+^ and CD8^+^ T cells to the lung of resveratrol-treated tumor bearing mice. The enhanced CD8^+^ T cell activity and Th1 immune responses induced by resveratrol might be related to the downregulated PD-1 expression on pulmonary CD8^+^ T cells and CD4^+^ T cells. Resveratrol also converted macrophages to M1 phenotype in the lungs of tumor bearing mice [200]. In addition to regulation of PD-1 expression on tumor-infiltrating T cells, resveratrol can function as a direct inhibitor of α-glucosidase/α-mannosidase that modulates *N*-linked glycan decoration of PD-L1, thereby promoting the ER retention of a mannose-rich, abnormally glycosylated form of PD-L1. By interfering with PD-L1 trafficking, resveratrol impeded PD-L1 moving to the MDA-MB-231 cell plasma membrane and then enhanced antitumor T-cell immunity. As predicted by computer modeling, resveratrol also directly binds to PD-L1 surfaces to induce PD-L1 dimerization and block PD-1 binding [201]. It has been found that PD-L1 glycosylation is required for PD-L1/PD-1 ligation. Targeting glycosylated PD-L1 blocks PD-L1/PD-1 interaction and promotes PD-L1 internalization and degradation, resulting in the efficient eradication of 4T1 TNBC tumor growth and elongation of mouse survival [202]. Paclitaxel induces chromosome mis-segregation on multipolar spindles during mitosis, which potentially activates cyclic GMP-AMP synthase (cGAS) and may induce a type I interferon response reliant on the stimulator of interferon genes (STING) pathway. A clinical study reported that elevated baseline cGAS expression significantly correlated with treatment response in patients receiving microtubule-targeting agents in combination with ICIs. Some TNBC patients with high levels of tumor cGAS had durable responses to combination therapy (exceeding 20 months). By contrast, patients with tumors expressing lower levels of cGAS had disease progression several months after initiation of therapy. This indicates that microtubule-targeting agents sensitize tumors that express cGAS to ICIs in TNBC [203].

The TME can promote cancer cell transition into the mesenchymal state, since many cells such as cancer-associated fibroblasts, TAM, MDSCs, and Treg cells can secrete TGFβ to induce the expression of EMT-inducing transcription factors (EMT-TFs), including ZEB, SNAIL, and TWIST [204]. EMT is a reversible cellular process that transiently places epithelial cells into mesenchymal cell states. Epithelial cells displaying apical–basal polarity are held together by tight junctions, adherent junctions, and desmosomes, which help maintain cell polarity and the epithelial state [205]. As tumor progression proceeds, neoplastic cells gradually acquire the mesenchymal state, which is promoted by EMT-TFs. EMT-TFs inhibit the expression of epithelial markers (e.g., E-cadherin, occludin, and claudins) but activate the expression of mesenchymal markers (e.g., N-cadherin, vimentin and fibronectin). EMT development includes the destruction of intercellular connections and cell–matrix adhesive interaction, ECM breakdown, and cleavage of basement membrane (BM) components by MMP activity modulation. That is why EMT gives cancer cells the advantage to migrate to secondary sites [205]. Furthermore, EMT is an important mechanism by which tumors become multidrug-resistant [206,207]. EMT-TFs promote resistance to oxaliplatin-based and cisplatin-based chemotherapies in breast, ovarian, colon, and pancreatic cancers by regulating genes involved in cell death and cancer stem cell maintenance [208,209]. In addition to conferring chemoresistance, EMT can confer resistance to immunotherapy. Thus, phytoagents with the ability to reverse or inhibit EMT might help overcome chemoresistance or suppress tumor metastasis.

Resveratrol inhibited the mammary tumor growth and lung metastasis of MDA-MB-231 human breast cancer in a xenograft-bearing mouse model and inhibited MDA-MB-231 cell migration and EMT in vitro [210]. Resveratrol also enhanced antitumor effects and reduced body weight loss and kidney function impairment induced by cisplatin in MDA-MB-231 xenograft-bearing mice. Resveratrol combined with cisplatin synergistically inhibited the viability, cell invasion, and migration abilities through inhibition of EMT of MDA-MB-231 cells. The combination treatment significantly reduced the expressions of p-AKT, p-PI3K, Smad2, Smad3, p-JNK, p-ERK, and NF-κB in tumor tissues, all of which are signaling molecules involved in TGF-β1-induced EMT [211].

Cellular communication network factor 5 (CCN5) inhibits the stemness, reverses the EMT process in breast cancer cells [212], and activates ER-α in TNBC cells [213]. Therefore, targeting TNBC by activating CCN5 would be an ideal strategy. EGCG activates CCN5 to inhibit human MDA-MB-231 cell sphere-forming ability via reversing the stemness of TNBC cells and EMT process in vitro. EGCG upregulates CCN5 expression in the tumor and effectively delays tumor growth in the human TNBC xenograft model [214].

### 4.3. Effects on Exosomes, Epithelial-Mesenchymal Transition and Extracellular Matrix

Tumor-derived exosomes are vesicles with the size of a virus (~100 nm) carrying a cargo of DNA, RNAs or proteins with immunomodulatory activity on the surrounding immune cells. Exosomes isolated from tumor cell supernatants or cancer patients show a rich expression of Fas ligand (FasL), PD-L1, IL-10, TGF-β, tumor-associated antigens, and ectoenzymes engaged in the adenosine pathway (CD39 and CD73), which all contribute to immunoevasion [215]. Certain phytoagents with the potential to change immunosuppressive exosomes into immunostimulatory ones might help strengthen anticancer immunity. EGCG treatment decreased the levels of colony stimulating factor 1 (CSF-1) and CCL-2 within the tumor and hence reduced the infiltration of TAM-like M2 macrophages in the 4T1-bearing mice. EGCG also upregulated miR-16 in tumor cells, which can be transferred to the TAM via exosomes and inhibited TAM infiltration and M2 polarization [216].

DET and DETD-35, phytosesquiterpene lactone analogs, were observed to effectively suppress human MDA-MB-231 cell activity and tumor growth in mice by inducing ROS production and increasing cytosolic calcium level and caused mitochondrial dysfunction and structural change of the treated TNBC cells [217]. Notably, DET/DETD-35 treatments were observed to induce oxidative stress-induced TNBC cell releasing exosomes, which showed antiproliferative activity against the parental MDA-MB-231 cells. Quantitative proteome analysis of TNBC cell-secreted exosomes showed that DET and DETD-35 affected several exosomal proteins participating in biological mechanisms such as oxidative stress and decreased transmembrane potential of mitochondria; these two compounds also attenuated the expression of proteins related to cell migration/adhesion and angiogenesis [217].

Cancer metastasis consists of a complex cascade of events, which ultimately allow for tumor cell extravasation and seeding in ectopic environments. For cancer cells to develop metastasis, they must break through and dissolve ECM and BM. The degradation of the pericellular BM and ECM is catalyzed by several classes of ECM-degrading enzymes, including MMPs [218]. Phytochemicals with the potential to inhibit MMP might prevent cancer metastasis. Resveratrol suppression of 4T1 cancer metastasis to the lung in vivo involved a decrease in MMP-9 [219]. Berberine diminished tumor growth and lung metastasis of TNBC cells in vivo through inhibition of TGF-β1 expression. In vitro, berberine also inhibited MMP-2 expression, colony formation, and cell migration in the wound-healing assay of BT549 and MDA-MB-231 cells [220]. Berberine suppressed MDA-MB-231 tumor growth in vivo and increased caspase-9 level in xenograft tumors [221]. DET abolished TNFα-induced MMP-9 enzyme activity and expression, and NF-κB activation in TS/A tumor cells [59]. On the other hand, when paclitaxel was combined with E-3810, a dual inhibitor targeting tyrosine kinases in VEGF receptors and fibroblast growth factor receptor, the combination showed a striking activity with complete, lasting tumor regressions on TNBC MDA-MB-231 and MX-1 xenograft [222]. This antitumor effect resulted from the cytotoxic/apoptotic effect of paclitaxel on tumor cells (activating caspase-3/7 activity) and a proteolytic remodeling of the extracellular matrix by regulation of collagen IV disposition and MMP-9 caused by E-3810 [222]. Effects of phytoagents on immune response and on the regulation of TNBC growth or metabolisms in preclinical animal models are summarized in Table 1.

## 5. Highlights of Some Clinical Trial Studies for Plant-Derived Drugs against Breast Cancers

TNBC is a molecularly heterogeneous disease; therefore, combinational therapy is encouraged as the standard-of-care treatment option [223]. Plant-derived paclitaxel, as a microtubule-targeting agent, was first identified in the 1960s [73] and has been well-characterized as standard first-line chemotherapy for advanced and metastatic breast cancer patients. Paclitaxel has often been embedded in novel regimes or therapeutic strategies with conventional therapy, targeted therapy, immunotherapy, or developed nanomedicinal delivery approaches. Moreover, paclitaxel has been proposed to be combined with other chemotherapy drugs, targeted therapies, and neoadjuvants with different mechanisms to provoke the antitumor effects. As of December 2021, there were more than 100 paclitaxel-related clinical trials that were recruiting, enrolling by invitation, or activated, and more than 50 paclitaxel-related clinical trials that were completed for testing potential treatments for TNBC patients. When paclitaxel was combined with atezolizumab (anti-PD-L1), the overall survival benefit was observed in PD-L1 tumor-infiltrating immune cells-positive patients [17]. However, combination treatment of iniparib (anti-PARP) with paclitaxel did not increase relevant antitumor activity in early TNBC [224]. This could be due to the optimal dose and treatment schedule of iniparib that had not been adequately defined in the phase I trial [224]. Another clinical study showed that the objective response rate and progression free survival were similar in the treatment of paclitaxel alone or the combination of paclitaxel plus tigatuzumab, a humanized agonistic monoclonal antibody directed against human tumor necrosis factor-related apoptosis-inducing ligand receptor 2 (also known as death receptor 5 (DR5)). However, prolonged progression-free survival in few patients was observed with paclitaxel plus tigatuzumab therapy, which might be associated with ROCK1 gene pathway activation [225]. Cytotoxic chemotherapies known to induce immunogenic tumor cell death, such as paclitaxel, can sensitize tumors to checkpoint blockade therapy [17]. Overall, paclitaxel has been often broadly proposed in combination with other new class of interventions for TNBC patients.

Combining agents with noncytotoxic treatments of complementary mechanism of action may improve outcomes of chemotherapy and overcome chemoresistance without significantly increased toxicity. Artemisinin has demonstrated its promising antimalaria effect and was awarded a Nobel prize in 2015. In recent decades, there has been increasing attention paid to the anticancer activity of artemisinin, and this has been demonstrated in animal studies. Although the safety use of artemisinin in human subjects has been proved in clinical studies [226,227,228,229] and empirical evidence from Chinese medicinal history, the actual benefit from anticancer activity of artemisinin on cancer patients require to be further validated. A clinical study, ARTIC-M33/2, was designed for the pharmacokinetics [71] and short-term [72,73] and long-term toxicity [74] studies. The pharmacokinetics study results suggested that therapeutic use of artesunate can be monitored by dihydroartemisinin in saliva [71]. Additionally, four weeks of short-term use of oral artesunate (up to 200 mg/d) was safe and well tolerated, although 3 patients of a total 23 patients were observed with a temporary dose-limiting vertigo [72,73]. After up to 1115 days of cumulative treatment, thirteen patients did not exhibit any major safety concerns, which provided an excellent safety profile on long-term treatment patients with metastatic breast cancer [74].

On the other hand, P2Et extract has been proved its strong antitumor effects via directly targeting tumor cells or through immune-activation in various translational animal models [104,107,191,192,193], which make P2Et extract move on to an ongoing clinical trial study for its potential clinical use in patients with breast cancer (NCT05007444). Minnelide Capsules (triptolide) has an ongoing clinical study for evaluating its safety, pharmacokinetic, and pharmacodynamics in patients as a monotherapy or in combination with paclitaxel (NCT03129139). Targeting topoisomerase may have advantages over inhibiting microtubule in TNBC patients, given that altered DNA repair pathways are common in TNBC. Therefore, clinical trials using sacituzumab govitecan [82] or etoposide [90] are efficient to treat metastatic breast cancer patients. In 2016, sacituzumab govitecan was even assigned as a “breakthrough therapy” by the U.S. FDA for the treatment of patients with metastatic TNBC. Moreover, confirmed objective responses were also noted in patients who had previously received PD-1 or PD-L1–based therapy, suggesting the potential usefulness of combination therapy [83]. Completed clinical trials regarding the effects of phytoagents used alone or in combination for TNBC patients are summarized in Table 2. 

## 6. Current Challenges and Future Prospects for Development of Phytoagents for TNBC Therapy

Historically, plant-derived natural products have been a bedrock of drug discovery. It is estimated that a significant proportion of current medical drugs originated from plant natural products. Paclitaxel, camptothecin, and etoposide are well-known examples of the most commonly used chemotherapy drugs. However, these plant-derived anticancer drugs cannot meet clinical demands due to their commonly induced drug resistance and unpleasant side effects. Current research therefore aims at finding complementary and modern pharmacotherapies. To treat breast cancer, the development of novel pharmacotherapies is particularly urgent for TNBC patients, because TNBC is the most aggressive, metastatic, and highly challenging breast cancer subtype worldwide. Recently, several strategies aiming at novel targets have been introduced to try to maximize the efficacy of the therapeutic outcome for TNBC, including regulation of metabolic changes and the tumor microenvironment to combat development of chemoresistance and recurrence of TNBC. Combinations of plant-derived natural products with conventional anticancer therapies have shown promising outcomes and gained considerable attention from scientists worldwide due to enhancement of anticancer efficacy, reduction in adverse effects from conventional therapy, or/and prolonging survival time in pre-clinical animal models [230]. Therefore, several plant-derived natural products, such as curcumin, laetrile, mistletoe, and selected vegetables/Sun’s Soup [231] are included as “Integrative, Alternative, and Complementary Therapies” in the National Cancer Institute’s comprehensive source of cancer information for physician data query. In addition, certain potential plant-derived natural products are currently undergoing clinical trials, including sulforaphane (NCT03934905), peppermint essential oil (NCT04478630), and Guaraná (NCT00615316) for reduction in nausea and vomiting, radiation-related fatigue, and doxorubicin-associated cardiac dysfunction for breast cancer patients. A large number of validation studies at clinical trials await confirmation of their efficacy for TNBC patients. Utilization of systematic approaches to efficiently and robustly identify, validate, and characterize bioactive plant-derived natural products as adjuvants or as a novel class of therapeutics for TNBC patients is a crucial and important goal for the toughest breast cancers in women.

**Table 1:** In vitro bioactivities, molecular mechanisms, and preclinical animal studies concerning the anticancer effect of bioactive phytoagents/phytoextracts.

Compound Alone or in Combination	Molecular Mechanisms/Targets	Preclinical Animal Model	Ref.
***Terpenoids***			
***Monoterpenoids*** **Thymoquinone**	↑p-p38, ROS↑PARP cleavage, TUNEL ↓XIAP, survivin, Bcl-xL, Bcl-2 ↓Ki67, tumor growth	Subcutaneous injection of MDA-MB-231 in nude mice	[56]
**Thymoquinone in liposomal nanoparticles**	↓eEF-2K, Src/FAK, Akt/NF-κB ↑miR-603, ↓cell proliferation ↓migration and tumor growth	Orthotopic injection of MDA-MB-231 and MDA-MB-436 in nude mice	[55]
**Thymoquinone-loaded, hyaluronic acid-conjugated copolymer nanoparticles**	↑miRNA-361 ↓Rac1, RhoA, VEGF-A ↓vascularization	Orthotopic injection of 4T1 tumor model in BALB/c mice	[57]
**Thymoquinone + Doxorubicin**	↓XIAP, surviving, Bcl-xL, Bcl-2 ↑TUNEL ↓Ki67, tumor growth	Subcutaneous injection of MDA-MB-231 in nude mice	[56]
**TQFL12**	↑stabilize AMPKα ↑p-acetyl-CoA, apoptosis ↓cell growth, migration, invasion	Orthotopic injection of 4T1 tumor model in BALB/c mice	[58]
***Sesquiterpene lactones*** **DET/DETD-35**	↑G_2_/M cell-cycle arrest, cell apoptosis ↓migration, invasion, motility ↑cytoplasmic vacuolation ↑exosome release/affect exosomal proteins ↑p-ERK, p-JNK, p-p38 ↑ubiquitinated protein accumulation ↑ER stress-mediated paraptosis and apoptosis, ROS, LC3,	Orthotopic/lung metastatic MDA-MB-231 tumor model in NOD/SCID mice	[63,217]
**DETD-35 + paclitaxel**	↓VEGF, COX-2, Ki67 ↑caspase-3 ↓metastatic pulmonary foci	Lung metastatic MDA-MB-231 tumor model in NOD/SCID mice	[62]
**Artemisinin**	↓TGF-β mRNA levels, MDSC, Treg cells ↑TNFα mRNA levels, Tbet ↑CD4^+^ IFN-γ^+^ T cells ↑cytotoxic T lymphocytes ↓tumor growth ↑survival	Orthotopic 4T1 tumor model in BALB/c mice	[189]
**Artemisinin** **Artemisinin-loaded biotin-PEG-PCL polymers**	↑BAX, ratio of BAX/Bcl-2 ↓tumor growth ↑BAX, ratio of BAX/Bcl-2;↓Bcl-2 ↓tumor growth	4T1 tumor model in BALB/c mice	[67]
**Artesunate + irinotecan in phosphatidylcholine-based liposomes**	↓tumor growth	4T1 tumor model in BALB/c mice	[70]
**Dihydroartemisinin + docetaxel in a pH-sensitive nanoparticle delivery system**	↑ROS, p53, cytochrome c release;↓Bcl-2 ↑caspase-3 ↓mitochondrial membrane potential ↓tumor growth;↓metastasis	Orthotopic injection of 4T1 tumor model in BALB/c mice	[66]
**Dihydroartemisinin** **Dihydroartemisinin+ docetaxel in disulfide-linked nanoparticle delivery system**	↑early apoptosis ↑cell cycle arrest ↓tumor growth ↑cell cycle arrest ↑early apoptosis ↑sustained release, circulating time ↓migration, tumor growth ↑prolonged survival	Orthotopic injection of 4T1 tumor model in BALB/c mice	[65]
***Diterpenoids*** **Paclitaxel** **Paclitaxel + tyrosine kinases inhibitor (E-3810)**	↑caspase-3/7 activity ↑ECM remodeling, ↓tumor growth ↑caspase-3/7 activity ↑ECM remodeling, MMP-9 ↓tumor growth	Subcutaneous injection of MDA-MB-231 and MX-1 tumor model in nude mice	[222]
**Triptolide**	↓HMGB1, TLR4, p-NF-κB ↓cell viability, clonogenic ability ↓Tumor growth	Subcutaneous injection of MDA-MB-231 tumor model in nude mice	[76]
	↓CD206, Arginase-1, CD204 ↓M2 TAM ↓anti-inflammatory cytokines ↓tumor growth	Orthotopic injection of 4T1 tumor model in BALB/c mice	[190]
	↓VEGF-A, angiogenesis ↓ERK1/2, HIF1-α ↓tumor growth, tumor cell proliferation	Orthotopic injection of MDA-MB-231 tumor model in nude mice	[196]
***Polyphenols***			
**Curcumin**	↑miR181b ↓CXCL-1 and CXCL-2 ↓lung metastasis	Intracardiac injection of human MDA-MB-231 cells in immunodeficient mice	[184]
	↓Ki67 ↓VEGFR2/3 ↓micro-vessel density	Subcutaneous injection of human MDA-MB-231 cells in immunodeficient mice	[194]
**Curcumin (before tumor inoculation) + *Listeria*-Mage-b vaccine (therapeutic immunization)**	↓IL-6 by MDSCs in tumor/blood ↑IL-12 by MDSCs in blood ↑IFNγ by CD4^+^ and CD8^+^ T cells in blood	Orthotopic injection of 4T1 cells in BALB/c mice	[182]
**Curcumin + metformin**	↑tumor apoptosis ↑Serum IL-4	Subcutaneous injection of EMT6/P cells in BALB/c mice	[183]
**Curcumin + arabinogalactan**	↓Ki67 ↑p53	Subcutaneous injection of 4T1 cells in BALB/c mice	[30]
**Curcumin + calcitriol**	↓micro-vessel density	Subcutaneous injection of MBCDF-T cells in nude mice	[195]
**Meriva administered after cryoablation**	↓IL-6	Orthotopic injection of 4T1 cells in BALB/c mice	[185]
**Resveratrol**	↓lung nodules ↓plasma MMP-9	Intravenous injection of 4T1 cells to develop lung metastasis in BALB/c mice	[219]
	↓MMP-2, MMP-9, vimentin, snail1, slug ↑*E*-cadherin	Orthotopic injection of human MDA-MB-231 cells in a xenograft model	[210]
	↑IFNγ and IL-2, M1 TAM in the lung ↑lung-filtrating CD4^+^ and CD8^+^ T cells ↑perforin/granzyme on splenic CD8^+^ T cells ↓PD-1 on pulmonary CD4^+^ and CD8^+^ T cells	Intravenous injection of 4T1 cells to develop lung metastasis in BALB/c mice	[200]
	↓Bregs, TGFβ, Treg	Orthotopic injection of 4T1 cells in BALB/c mice	[186]
	↓FASN expression ↓lipid synthesis	Orthotopic injection of human MDA-MB-231 cells in nude mice	[130]
	a 5-LOX inhibitor ↓COX-2 and MMP-9 expression	Rats treated with DMBA to induce mammary cancer	[43,44]
	↑LC3-II, Beclin1 and Atg 7 in BCSCs ↓Wnt/β-catenin signaling pathway in BCSCs	Orthotopic injection of human SUM159 cells in NOD/SCID mice	[46]
**Resveratrol + tamoxifen**	↓acetylated STAT3 ↑*ER-α* gene expression	Subcutaneous injection of human MDA-MB-231 cells in nude mice	[42]
**Resveratrol + cisplatin**	↓p-AKT, p-PI3K, Smad2, Smad3, p-JNK, p-ERK, and NF-κB in tumor tissues	Orthotopic injection of human MDA-MB-231 cells in a xenograft model	[211]
**EGCG**	↓CD44^+^ BCSCs ↓VEGF ↓MMP-2 ↑Caspase-3	Rats treated with 7,12 dimethylbenzanthracene (DMBA) to induce mammary cancer	[48]
	↓RNA levels of *cyclin D1 (CCND1), RHOC, fibronectin (FN1), E-cadherin (CDH1), vimentin (VIM)* and *BCL-XL* ↓VEGF expression ↓tumor sphere formation	Orthotopic injection of human ALDH-positive SUM-149 cells in NOD/SCID mice	[49]
	↓CSF-1, CCL-2, IL-6, and TGFβ ↓infiltration of M2 TAM	Subcutaneous injection of 4T1 cells in BALB/c mice	[216]
	↓tumor glucose and lactic acid levels ↓tumor VEGF	Subcutaneous injection of 4T1 cells in BALB/c mice	[121]
	↑CCN5 expression ↓EMT, stemness	Subcutaneous injection of human MDA-MB-231 cells in nude mice	[214]
**EGCG + taxol**	↑tumor apoptosis ↓tumor GRP78, JNK phosphorylation	Murine breast 4T1 cells in BALB/c mice	[153]
**EGCG + cetuximab**	↓FASN activity	Orthotopic injection of sensitive and chemoresistant TNBC cells	[129]
***Alkaloids***			
**Sacituzumab Govitecan + PARP inhibitors (olaparib or talazoparib)**	↑γ-H2AX	Subcutaneous injection of human *BRCA1/2*-mutated or—wild-type TNBC cells in nude mice	[86]
**Camptothecin + doxorubicin**	↓M2-like TAMs	Orthotopic injection of 4T1 cells in BALB/c mice	[187]
**Camptothecin-loaded nanoparticle displaying cetuximab**	↓Ki67	Orthotopic injection of bone-metastatic MDA-MB-231 cells in NSG mice	[77]
**bevacizumab + CRLX101 (a nanoparticle–drug conjugate containing camptothecin)**	↓HIF1α ↓hypoxia	Orthotopic injection of highly aggressive variant MDA-MB-231 cells (LM2-4) in SCID mice	[79]
**Etoposide + TMU-35435**	↑LC3, γ-H2AX, caspase-3	Orthotopic injection of 4T1 cells in BALB/c mice	[91]
**Etoposide + TRAIL**	↑DR5 expression ↑PARP, caspases and p53 expressions	Orthotopic injection of human MDA-MB-231 cells in a xenograft model	[93]
**Berberine**	↓TGF-β1 ↓MMP-2	Orthotopic injection of MDA-MB-231 or 4T1 cells in mice	[220]
	↓Ki67 ↑caspase-9	Orthotopic injection of MDA-MB-231 cells in nude mice	[221]
	Berberine binds to VASP Secondary structure of VASP changes ↓actin polymerization	Subcutaneous injection of human MDA-MB-231 cells in nude mice	[98]
	↓NF-κB, IL-1β, IL-6 and TNFα ↓PCNA	Rats treated with DMBA to induce mammary cancer	[232]
**Berberine + anti-DR5 antibody**	↑caspase-3 ↑PARP	Orthotopic injection of 4T1 cells in BALB/c mice	[99]
**co-loaded liposome of berberine and doxorubicin**	↓cardiotoxicity ↓tumor	Subcutaneous injection of 4T1 cells in BALB/c mice	[188]
***Plant extracts/other phytocompounds***			
**Sulforaphene**	↓cell proliferation ↓cyclin B1, Cdc2 ↑G2/M phase arrest, Egr1	Orthotopic injection of MDA-MB-453 tumor model in nude mice	[100]
	↓CRIPTO-1/TDGF1 ↓CRIPTO-3/TDGF1P3 ↓Nanog, ALDH1A1, Wnt3, Notch 4	Orthotopic injection of MDA-MB-231 tumor model in nude mice	[101]
**Sulforaphene** **Sulforaphene + doxorubicin**	↓cell growth, HDAC6;↑autophagy ↑membrane translocation ↑acetylation modification of PTEN synergistic inhibition on MDA-MB-231 xenografts growth.	Orthotopic injection of MDA-MB-231 tumor model in nude mice	[102]
**Sulforaphene** **Sulforaphene + docetaxel**	↓NF-κB p65 translocation;↓p52 ↓mammosphere formation ↓taxane-induced ALDH^+^ cell enrichment ↓primary tumor volume ↓secondary tumor formation	Orthotopic injection of SUM149 tumor model in NOD/SCID mice	[103]
**P2Et** **(*Caesalpinia spinosa* extract)**	↑mitochondrial membrane potential loss ↑phosphatidylserine externalization ↑caspase 3 activation, IL-6, MCP-1 ↑DNA fragmentation ↓clonogenic capacity of 4T1 cells ↓tumor growth, spleen metastasis	Orthotopic injection of 4T1 tumor model in BALB/c mice	[104]
	↑calreticulin, ATP secretion ↑HMGB1 translocation ↑IL-2, TNFα, IL-4, IL-5 ↑IFNγ-producing CD4^+^ and CD8^+^ T cells	Orthotopic injection of 4T1 tumor model in BALB/c mice	[191]
	↓cell viability, proliferation ↓tumor growth	Orthotopic injection of MDA-MB-468 tumor model in NSG mice	[107]
**Prophylactic therapy of P2Et**	↑CD4^+^ T, CD8^+^ T, NK, DC ↑Treg, MDSC, plasma IL-6	orthotopic injection of 4T1 tumor model in BALB/c mice	[192]
**P2Et + antiPD-L1**	↓tumor growth, granulocytes	orthotopic injection of 4T1 tumor model in BALB/c mice	[193]

**Table 2:** Clinical studies concerning the anticancer effects of monotherapy of plant-derived drugs or in combination with other clinical drugs.

Compound Alone or in Combination	Molecular Mechanisms/Targets	Treatment Results	Phase; Intervention	Ref.
***Terpenoids***				
**Artesunate** **as add-on therapy**	Anticancer ↓TGF-β mRNA levels, MDSC, Treg cells ↑TNFα mRNA levels, Tbet ↑CD4^+^ IFN-γ^+^ T cells ↑cytotoxic T lymphocytes ↓tumor growth ↑survival	The pharmacokinetics of artesunate and its metabolites—dihydroartemisinin was well described by a combined drug-metabolite model. The saliva sampling for artesunate monitoring of dihydroartemisinin was suggested.	ARTIC-M33/2 Metastatic breast cancer patients (phase I, *n* = 23) 100, 150, or 200 mg oral artesunate daily as add-on therapy to their guideline-based oncological therapy.	[189,226]
	Anticancer ↓TGF-β mRNA levels, MDSC, Treg cells ↑TNFα mRNA levels, Tbet ↑CD4^+^ IFN-γ^+^ T cells ↑cytotoxic T lymphocytes ↓tumor growth ↑survival	The continuous intake of artesunate for 4 weeks in doses up to 200 mg daily was well tolerated in test patients. However, a temporary dose-limiting vertigo was observed in three patients.	ARTIC-M33/2 Metastatic breast cancer patients (phase I, *n* = 23) 100, 150, or 200 mg oral artesunate daily as add-on therapy to their guideline-based oncological therapy.	[189,227]
	Anticancer ↓TGF-β mRNA levels, MDSC, Treg cells ↑TNFα mRNA levels, Tbet ↑CD4^+^ IFN-γ^+^ T cells ↑cytotoxic T lymphocytes ↓tumor growth ↑survival n	200 mg/d are recommended for phase II/III trials.	ARTIC-M33/2 Metastatic breast cancer patients (phase I, *n* = 23) 100, 150, or 200 mg oral artesunate daily as add-on therapy to their guideline-based oncological therapy.	[189,228]
	Anticancer ↓TGF-β mRNA levels, MDSC, Treg cells ↑TNFα mRNA levels, Tbet ↑CD4^+^ IFN-γ^+^ T cells ↑cytotoxic T lymphocytes ↓tumor growth ↑survival	In 13 patients with metastatic breast cancer, up to 200 mg/d long-term oral artesunate in up to 1115 cumulative treatment days (cumulative doses up to 167.3 g) did not result in any major safety concerns.	ARTIC-M33/2 Metastatic breast cancer patients (phase I, *n* = 23) 100, 150, or 200 mg oral artesunate daily as add-on therapy to their guideline-based oncological therapy.	[189,229]
**Paclitaxel + Atezolizumab (anti-PD-L1)**	Targeting microtubule and PD-L1	The median OS of 25.4 months (19.6–30.7 months) with Paclitaxel + Atezolizumab (*n* = 185) and 17.9 months (13.6–20.3 months) with Paclitaxel + Placebo + nP (*n* = 184) in PD-L1 IC-positive population (*n* = 369).	Metastatic TNBC Patients (Phase III); nab-paclitaxel (100 mg/m^2^ of body surface area on days 1, 8, and 15 of every 28-day cycle) was combined with either placebo (*n* = 451) or atezolizumab (840 mg on days 1 and 15 of each cycle, *n* = 451).	[17]
**Paclitaxel + iniparib (PARP inhibitor)**	Targeting microtubule and PARP	pCR rate was similar among the three arms (21, 22, and 19% for PTX, PWI, and PTI, respectively). pCR in breast and axilla (21, 17, and 19%); best overall response in the breast (60, 61, and 63%); and breast conservation rate (53, 54, and 50%).	141 TNBC patients with Stage II-IIIA TNBC were randomly assigned to receive paclitaxel (80 mg/m^2^, d1; *n* = 47) alone (PTX) or in combination with iniparib, either once-weekly (PTW (11.2 mg/kg, d1; *n* = 46) or twice-weekly (PTI) (5.6 mg/kg, d1, 4; *n* = 48) for 12 weeks.	[224]
**Paclitaxel + Tigatuzumab (anti-DR5)**	Targeting microtubule and DR5 ROCK1 gene pathway activation	3 CR, 8 PR; 1 almost CR, 11 SD, and 17 PD in the combination arm (ORR, 28%). No CRs, 8 PRs, 4 SDs, and 9 PDs in the Paclitaxel arm (ORR, 38%). There was a numerical increase in CRs and several patients had prolonged PFS in the combination arm.	TBNC patients (Phase II) A treatment cycle was defined as 4 weeks. Patients received intravenous nab- aclitaxel on days 1, 8, and 15 (100 mg/m^2^) at 28 days interval with (*n* = 39) or without (*n* = 21)) Tigatuzumab intravenously on days 1 and 15 of every cycle (10 mg/kg loading dose followed by 5 mg/kg every other week).	[225]
***Polyphenols***				
**Curcumin + docetaxel**	↓carcinoembryonic antigen ↓VEGF ↓P-glycoprotein (P-gp, MDR1)	Five patients had PR, and three patients had SD at least 6 w after the last cycle of treatment. ORR was up to 50%. no progressive disease was observed.	Metastatic breast cancer patients (phase I, *n* = 14) docetaxel (IV 100 mg/m^2^) every 3 week on day 1 for 6 cycles + curcumin (p.o. 500 mg/day) for 7 consecutive days by cycle	[155]
***Alkaloids***				
**Sacituzumab Govitecan**	Targeting TOP1 in the Trop-2-positive cells	Median PFS was 5.5 months, and median OS was 13 months. ORR was 33%.	refractory metastatic TNBC patients (phase I/II, *n* = 108) 10 mg/kg, intravenously on days 1 and 8 of each 21-day cycle	[83]
		Median PFS was 5.6 months, and median OS was 12.1 months. ORR was 35%.	Metastatic TNBC patients (phase III, *n* = 468) 10 mg/kg, intravenously on days 1 and 8 of each 21-day cycle	[82]
**Irinotecan + iniparib** **(PARP inhibitor)**	Targeting TOP1 and PARP	Median OS was 7.8 months. Intracranial RR was 12%, while intracranial CBR was 27%.	TNBC patients with new or progressive brain metastases (phase II, *n* = 37) Irinotecan 125 mg/m^2^ intravenously (IV) on days 1 and 8 of each 21 day cycle. Iniparib was dosed at 5.6 or 8 mg/kg IV on days 1, 4, 8, 11 of each 21 day cycle.	[81]
**Etoposide**	Targeting topoisomerase II	ORR was 25%. Nine patients achieved SD for more than 24 weeks and CBR was 53%. The median PFS and OS were 5 (range, 1.5–17.0 months) and 16 months (range, 3.0–51.0 months), respectively.	Metastatic breast cancer patients (phase I, *n* = 32) 60 mg/m^2^/d on days 1–10, followed by 11 days of rest	[88]
		Seven (9.3%) patients achieved PR and 29 (38.7%) had SD. Nine patients (12%) had SD for >24 weeks and the CBR was 21.3% (16/75). The median PFS was 4.5 (range, 1.3–7.7) months.	Metastatic breast cancer patients (phase II, *n* = 75) 60 mg/m^2^/d on days 1–10, followed by 11 days of rest	[89]
		Median PFS was 4 months, CBR was 18% (overall response rate 4%), and median OS from the start of treatment was 11 months.	Metastatic breast cancer patients (phase II, *n* = 75) 50 mg/day in 20-day cycles with 1-week of rest	[90]

Abbreviations: progression free survival (PFS); objective response rate (ORR); clinical benefit rate (CBR); overall survival (OS); partial response (PR); stable disease (SD); response rate (RR); pathologic complete response (pCR).

## Data Availability

Not applicable.
